# Tracing species replacement in Iberian marbled newts

**DOI:** 10.1002/ece3.7060

**Published:** 2020-12-15

**Authors:** Julia López‐Delgado, Isolde van Riemsdijk, Jan W. Arntzen

**Affiliations:** ^1^ Naturalis Biodiversity Center Leiden The Netherlands; ^2^ Institute for Biology Leiden University Leiden The Netherlands; ^3^ Present address: University of Leeds Leeds United Kingdom; ^4^ Present address: Institute for Evolution and Ecology, Tübingen University Leeds Germany

**Keywords:** amphibians, enclave, hybridization, introgression, secondary contact, *Triturus*

## Abstract

Secondary contact between closely related species can lead to the formation of hybrid zones, allowing for interspecific gene flow. Hybrid zone movement can take place if one of the species possesses a competitive advantage over the other, ultimately resulting in species replacement. Such hybrid zone displacement is predicted to leave a genomic footprint across the landscape in the form of asymmetric gene flow (or introgression) of selectively neutral alleles from the displaced to the advancing species. Hybrid zone movement has been suggested for marbled newts in the Iberian Peninsula, supported by asymmetric gene flow and a distribution relict (i.e., an enclave) of *Triturus marmoratus* in the range of *T. pygmaeus*. We developed a panel of nuclear and mitochondrial SNP markers to test for the presence of a *T. marmoratus* genomic footprint in the Lisbon peninsula, south of the enclave. We found no additional populations of *T. marmoratus*. Analysis with the software Structure showed no genetic traces of *T. marmoratus* in *T. pygmaeus*. A principal component analysis showed some variation within the local *T. pygmaeus*, but it is unclear if this represents introgression from *T. marmoratus*. The results may be explained by (a) species replacement without introgressive hybridization and (b) displacement with hybridization followed by the near‐complete erosion of the footprint by purifying selection. We predict that testing for a genomic footprint north of the reported enclave would confirm that species replacement in these marbled newts occurred with hybridization.

## Graphical abstract

Hybrid zone movement has been suggested in the marbled newts, supported by the discovery of a *Triturus marmoratus* enclave. Here, we tested for a genomic footprint of species replacement of the receding *T. marmoratus* by the advancing *T. pygmaeus*, employing 54 newly developed nuclear SNPs and one mitochondrial DNA marker. In the absence of genetic traces of *T. marmoratus* in *T. pygmaeus*, we propose two biogeographic scenarios of species replacement.

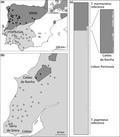

## 1 INTRODUCTION

Population genetic structure observed today has been shaped by demographic processes that operated in the past (Avise, 2004). Documenting present‐day allelic patterns may thus provide insight into species distribution dynamics over time (Avise et al., 1987; Crisci et al., 2003). Hybrid zones, where genetically distinct populations meet, mate and cross‐fertilize, may be particularly informative (Barton & Hewitt, 1985; Hewitt, 2001). These “natural laboratories” can be established when closely related species establish secondary contact, for example following range expansion from glacial refugia (Excoffier et al., 2009; Hewitt, 1988; Taberlet et al., 1998).

Moving hybrid zones may leave a specific spatial signature, in the form of a molecular genetic (or “genomic”) footprint away from the hybrid zone's current position (Scribner & Avise, 1993). When an advancing species spreads into a contact zone, neutral alleles may flow from the retreating to the invading taxon and introgression will be more pronounced in the advancing than in the retracting species (Barton, 1979; Moran, 1981). Asymmetric introgression may reflect neutral alleles left in the wake of the moving hybrid zone and eventually become geographically stable, because the genomic footprint is solely dependent on drift (Barton & Hewitt, 1985; Currat et al., 2008; Kulmuni et al., 2020). Hence, introgression patterns of selectively neutral traits can be used to reconstruct the geographical history of hybrid zones (Seixas et al., 2018; Wang et al., 2011; Zohren et al., 2016). Occasionally, the spatio‐temporal dynamics of species may result in the formation of enclaves (Buggs, 2007; Wielstra, 2019). Enclaves form when the population of one species is surrounded by populations of a mutually exclusive, competing species, therewith becoming genetically isolated from the remainder of the receding species' range (Arntzen, 1978; Littlejohn & Roberts, 1975). Enclaves can therefore illustrate historical species replacement, particularly in ground‐dwelling organisms with low dispersal capabilities, such as amphibians. The formation of enclaves may either take place with or without gene flow between the species involved.

**FIGURE 1:**
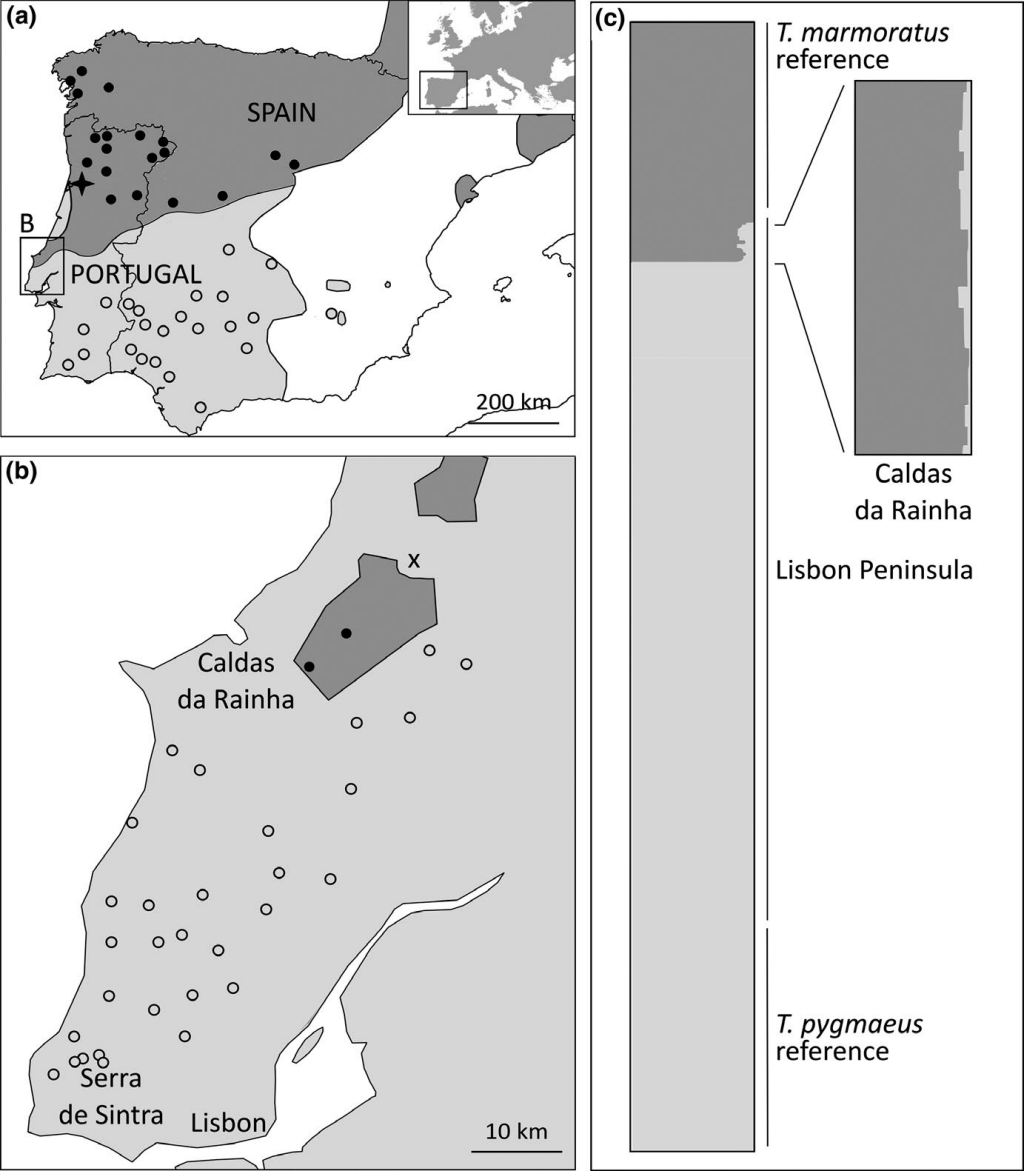
The Iberian Peninsula with records of *Triturus marmoratus* (solid round symbols) and *T. pygmaeus* (open round symbols). (a) Localities sampled for the evaluation of SNP marker diagnosticity. The boxed area includes the Lisbon Peninsula and the Caldas da Rainha area, with an inset of Europe for reference. The Aveiro Lagoon is shown by a four point star. (b) Localities sampled in the Lisbon Peninsula, with symbols as above. The continuous distribution with *T. marmoratus* in dark gray and *T. pygmaeus* in light gray is from Arntzen et al. (2009) and Arntzen (2018). Note the existence of a *T. marmoratus* enclave around Caldas da Rainha. The locality Juncal with both species and hybrids is shown by a cross. (c) Bar plot with individual admixture proportion (Structure *Q* scores) for *T. marmoratus* and *T. pygmaeus* reference populations and Lisbon Peninsula populations, with a detailed view of the Caldas da Rainha enclave (populations 1 and 2)

The Eurasian newt genus *Triturus* has been previously employed to investigate moving hybrid zones and enclave formation, for nine species at different spatial, environmental and phylogenetic settings (Arntzen & Wallis, 1991; Arntzen et al., 2014; Wielstra et al., 2017). The genus includes the northern marbled newt *Triturus marmoratus* (Latreille, 1880), which inhabits central and southern France as well as the northern part of the Iberian Peninsula, and the pygmy marbled newt *T. pygmaeus* (Wolterstorff, 1905), which occupies the southwestern parts of the Iberian Peninsula. These are sister species that engage in a hybrid zone spanning ca. 600 km across the west of the Iberian Peninsula (Figure 1a). We previously documented an enclave of *T. marmoratus* in the northwest of the Lisbon Peninsula, near the town Caldas da Rainha, composed of pure and introgressed populations (Figure 1b). Another remarkable spatial signature is the presence of *T. pygmaeus* in and along the coastal dunes, approximately from Caldas da Rainha to the Aveiro Lagoon (Figure 1). These observations suggest the northward competitive advance of *T. pygmaeus* over a stretch of more than 200 km, after which its range expansion was halted by the Rio Vouga estuary (Arntzen et al., in press).

The documented distribution pattern suggests that species replacement in Iberian marbled newts at the Portuguese Atlantic coast might take place through a moving contact zone, possibly leaving behind a tail of asymmetric introgression. We here test for the presence of genomic footprints of *T. marmoratus* in *T. pygmaeus* of the Lisbon Peninsula, south of the documented enclave, where we suspect that species turnover has taken place. For this study, we developed a panel of single‐nucleotide polymorphism markers (SNPs; Garvin et al., 2010) that were species diagnostic in a large reference sample, available from an earlier study (Arntzen, 2018). In addition, we modeled the mutual species' distributions from climatic conditions of the present day, the Mid‐Holocene and the Last Glacial Maximum, to investigate whether climate change would support the scenario of species replacement.

## 2 MATERIALS AND METHODS

### 2.1 Fieldwork, sampling, and DNA preparation

Fieldwork was carried out in 2018 and 2019 in the Lisbon Peninsula, where we searched for standing water bodies containing marbled newts. Twenty‐five localities with newt populations were found in an area spanning ca. 2,000 km^2^, ranging from the Tejo river in the east, the Tejo estuary in the south, and the Atlantic Ocean in the west. In the north, we sampled up to the town Caldas da Rainha where our survey adjoined the area previously investigated (Espregueira Themudo & Arntzen, 2007). We specifically included the Serra de Sintra mountains in the southwest of the Lisbon peninsula. This was because we suspected the (past) presence of *T. marmoratus* in these mountains, on account of (a) the distribution of *T. marmoratus* at higher altitudes than *T. pygmaeus* elsewhere along the species' mutual contact (Arntzen & Espregueira Themudo, 2008), and (b) a pilot study that revealed weak evidence for the presence of *T. marmoratus* genetic material at Sintra (Arntzen et al., in press).

Adult and larval newts were captured by dip netting or with funnel traps. To reduce sampling bias, for example, toward siblings from particular breeding pairs (Goldberg & Waits, 2010), we made an effort to include all accessible sections of the water bodies. Tail tip tissue samples were collected and stored in 96% ethanol. We also studied material from seven localities obtained earlier (Espregueira Themudo & Arntzen, 2007). DNA extraction of tissue samples followed the Kingfisher™ (Thermo Scientific) and DNeasy extraction kit (Qiagen) standard protocols.

### 2.2 SNP marker design

**FIGURE 2:**
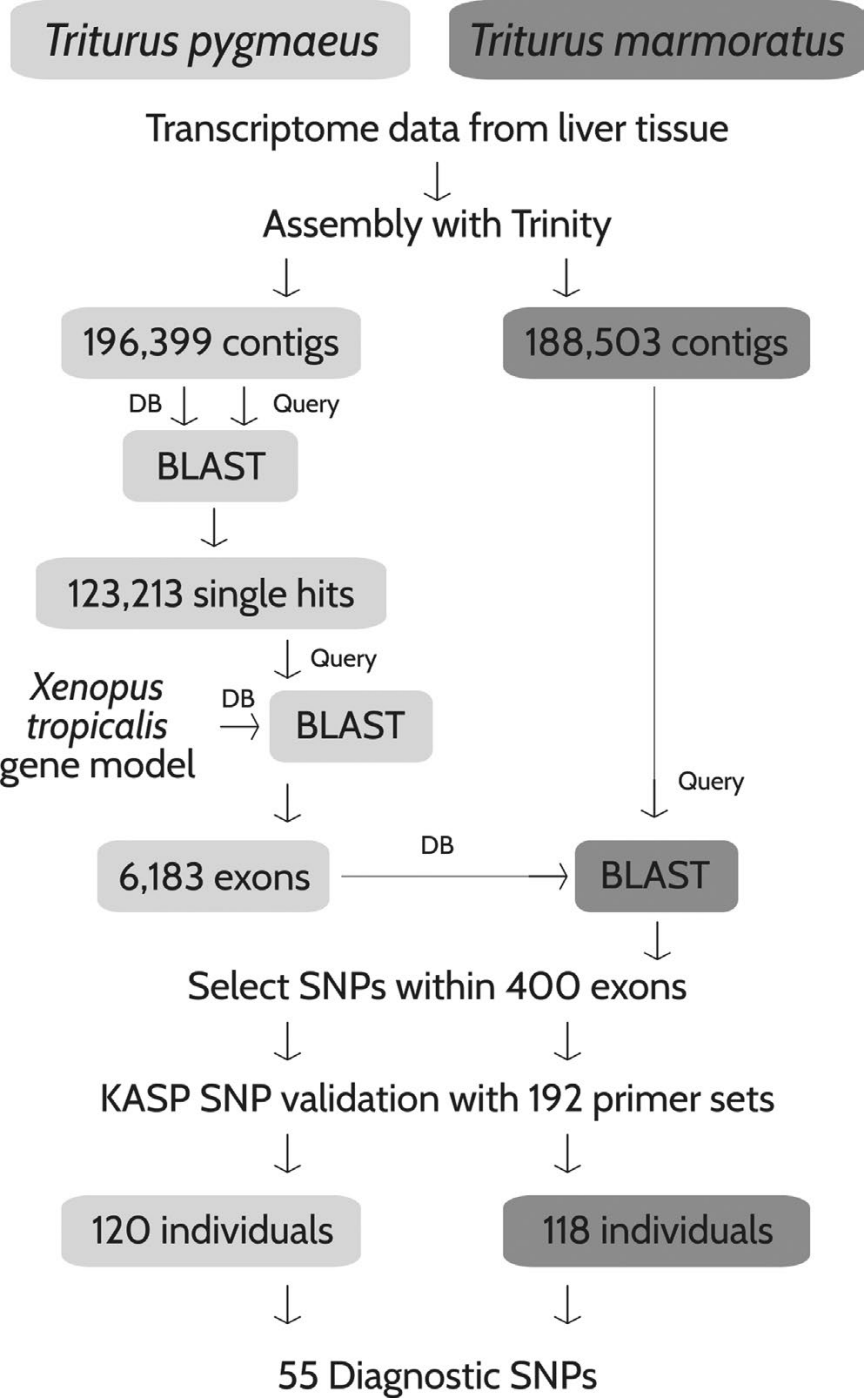
Schematic representation of the SNP design process, adapted from van Riemsdijk et al. (2019). For details see text

Transcriptome data for a male *T. marmoratus* collected from San Pedro da Cova (41.157 N, 8.496 W) and a male *T. pygmaeus* from Umbria, Serra de Monchique (37.335 N, 8.506 W), had been previously obtained through the Illumina HiSeq 2000 Sequencing System (Wielstra et al., 2019). The transcriptome libraries are available through the NCBI SRA at BioProject PRJNA498336 (https://www.ncbi.nlm.nih.gov/bioproject/PRJNA498336). Data filtering was performed with Trimmomatic v0.36 (Bolger et al., 2014) under default settings, except for the parameter “minlen” that was set to exclude read lengths below 60 base pairs. Accordingly, the number of paired reads dropped from 60.34 10^6^ to 46.21 10^6^ for *T. pygmaeus* and from 56.04 10^6^ to 42.67 10^6^ for *T. marmoratus*. Trinity v2.5.1 (Grabherr et al., 2011; Haas et al., 2013) was employed for *de novo* transcriptome assembly under default settings, resulting in 19.6 10^4^ contigs for *T. pygmaeus* and 18.8 10^4^ contigs for *T. marmoratus*. The transcriptome assembly was blasted against itself in order to identify and remove all hits occurring more than once, therewith discarding all contigs possibly representing paralogs (Altschul et al., 1990). Paralog filtering resulted in 12.3 10^4^ contigs for *T. pygmaeus* and 11.2 10^4^ contigs for *T. marmoratus*. An outline of the procedure is shown in Figure 2.

Single‐nucleotide polymorphism marker design followed the molecular inversion probes pipeline that encompasses advantages for targeted resequencing, including high specificity, a high level of multiplexing and no ascertainment bias (Hardenbol et al., 2003; Niedzicka et al., 2016). Given that the *T. pygmaeus* transcriptome had a higher number of contigs, the following exon selection step was carried out using this dataset. Gene models were constructed based on the *Xenopus tropicalis* (Gray, 1864) frog genome, available from Biomart ENSEMBL (genome version JGI4.2). To further remove potential paralogs, *T. pygmaeus* contigs were mapped to the gene models to select exons that map to single *X. tropicalis* genes; these unique hits, along with the exon boundary coordinates, were used to extract exon sequences from *T. pygmaeus* contigs.

A custom script further filtered exons according to their position within the *X. tropicalis* gene model to avoid sequences possibly containing 5′ or 3′ UTR. The blast between *X. tropicalis* gene models and *T. pygmaeus* contigs gave *T. pygmaeus* exons that occurred over two times, twice or once within the *X. tropicalis* models (Altschul et al., 1990). The filtering script prioritized exons found more than twice in the gene model (“priority 1”), followed by exons present twice (“priority 2”) and, finally, exons occurring only once (“priority 3”). Priority 1 exons were chosen as follows: whenever exons were present three times in the gene model, the exon located in the center was selected and if found over three times, an exon away from the extremes of the model was randomly selected. Priority 2 exons were designated by selecting the longest exon. Priority 3 exons were discriminated against as they provide no spatial information given their single presence within a gene model, with the risk that they would contain noncoding sequence. The model strictly selected exons based on length, selecting exons with a minimum of 100 base pairs to ensure sufficient length for primer attachment and a maximum of 400 base pairs, considering average exon length (Sakharkar et al., 2004). This noncoding sequence removal step resulted in 3,372 *T. pygmaeus* exons of which 42 exons were priority 1, 228 were priority 2 and 3,102 were priority 3.

*Triturus pygmaeus* exons were blasted against the *T. marmoratus* transcriptome contig database, with potential paralogs being manually removed from the BLAST output (Altschul et al., 1990). From the 3,372 blast hits, the 192 most suitable exons for SNP identification were scrutinized based on percentage identity, *e‐*value, number of gaps, and the potential presence of multiple similar sequences and/or identical sequences in different contigs. SAMtools (Li et al., 2009) was used to retrieve the 192 exon sequences for both species from the original transcriptome contig dataset. Genes were identified by blasting the sequences against the *X. tropicalis* database; nonetheless, three fragments could not be annotated as several gene models in this model organism are based on un‐annotated genes. SNPs were manually identified in Mesquite v3.40 (Maddison & Maddison, 2019) after performing a sequence alignment with Muscle v3.8.31 (Edgar, 2004).

### 2.3 SNP detection and validation

Single‐nucleotide polymorphism genotyping took place at the Institute of Biology Leiden/Naturalis SNP line facility using the Kompetitive Allele‐Specific PCR (KASP) genotyping system (LGC Genomics). KASP is a fluorescence‐based method determining the biallelic score of SNPs in uniplex assays. KASP is based on two allele‐specific primers with a final base complementary to one of the two potential SNPs and unique tail sequence (Semagn et al., 2014). The KASP master mix contains different fluorescent‐labeled primers that become activated during PCR cycles, with the fluorescent signal increasing as more fluorescent primers are incorporated during the thermocycling of the PCR reaction. Primers were designed using the Kraken software and ordered from Integrated DNA Technologies (Wood & Salzberg, 2014).

For SNP validation, we used 120* T. pygmaeus* and 118 *T. marmoratus* from 43 reference populations across the Iberian Peninsula, located outside the documented hybrid zone of these species (Arntzen, 2018; Figure 1, Table 1). The validation assay of 192 SNPs resulted in 147 markers being polymorphic, of which 81 were informative for species identification. For the Lisbon Peninsula, 354 individuals from 32 populations (25 new and seven previously studied; Table 2) were KASP genotyped for the 60 most promising nuclear SNPs. A further three primer sets were developed from the sequence information provided by Espregueira Themudo et al. (2012), for the nuclear genes β‐Fibrinogen intron 7 (BF), Calreticulin intron C (CC), and the Platelet‐derived growth factor receptor α intron 11 (PDG). Single‐nucleotide polymorphisms were considered species diagnostic for 54 nuclear markers with a low Cohen's kappa (1‐ĸ) > 0.9. Missing data amounted to 2.3%. We also studied the mitochondrial gene NADH dehydrogenase subunit 4 (ND4), with 1‐ĸ at unity and 2.0% missing data (Tables S1 and S2).

**TABLE 1:** Sample sites for the 43 populations used for SNP validation with population sizes (*N*). Population numbers R1–R19 are *Triturus marmoratus*, and R20–R43 are *T. pygmaeus*

Population	Locality name	Latitude	Longitude	*N*
R1	Ponte Maceira, Negreira, Spain	42.901	−8.703	2
R2	Mirador de la Curota, Spain	42.625	−8.960	2
R3	Rebordelo, Portugal	41.724	−7.123	2
R4	Gerez ‐ Vilar de Veiga, Portugal	41.703	−8.168	2
R5	Mogadouro, Portugal	41.331	−6.740	2
R6	San Pedro da Cova, Portugal	41.157	−8.495	2
R7	Mezio, Portugal	40.979	−7.885	2
R8	Castelo Mendo, Portugal	40.597	−6.933	2
R9	Muñotello, Spain	40.553	−5.015	2
R10	Nelas, Ponte Nove, Portugal	40.486	−7.836	2
R11	Serradilla del Llano, Spain	40.482	−6.319	2
R12	Geres ‐ Carris, Portugal	41.815	−8.046	10
R13	Cabreiras, Spain	42.438	−7.902	10
R14	Teixeira, Portugal	41.428	−6.475	16
R15	Vimioso, Spain	41.607	−6.498	9
R16	Fuenterrebollo, Laguna de Gómez, Spain	41.326	−3.926	16
R17	Santo Tomé del Puerto, Spain	41.200	−3.588	10
R18	Geres‐ Cabril, Portugal	41.714	−8.036	10
R19	Punta Moreiras, Spain	42.497	−8.876	15
R20	Buenasbodas nr. Belvis de la Jara, Spain	39.681	−4.889	2
R21	Los Yebenes ‐ estacion de Urda, Spain	39.418	−3.821	2
R22	Llerena ‐ Higueira de la Serena, Spain	38.668	−5.678	2
R23	Mitra II, Portugal	38.535	−8.000	2
R24	Orcera, Spain	38.320	−2.669	2
R25	Barrancos ‐ Aroche, Spain	38.069	−6.934	2
R26	Sao Domingos, Portugal	37.933	−8.511	2
R27	Cabra, Ermitage de la Virgen de la Siera, Spain	37.497	−4.368	2
R28	El Portil, Spain	37.245	−7.097	2
R29	Faro University, Portugal	37.050	−7.978	2
R30	Doñana, Spain	36.852	−6.394	2
R31	Los Barrios – Cacinas, Spain	36.189	−5.566	2
R32	Vale de Bispo – Sagres, Portugal	37.072	−8.904	8
R33	Mourao, Portugal	38.429	−7.312	8
R34	Granja, Spain	38.318	−7.262	8
R35	Belmez – Espiel, Spain	37.987	−4.785	8
R36	Villanueva de los Castillos ‐ El Granado, Spain	37.498	−7.303	8
R37	El Villar, Spain	37.210	−6.703	8
R38	Embalse de Aracena, Spain	37.924	−6.486	8
R39	Estacion de Belalcazar ‐ Sta Eufamia, Spain	38.654	−5.012	8
R40	Venta del Charco II, Spain	38.193	−4.281	8
R41	Cerro del Hierro, Spain	37.953	−5.623	8
R42	Llerena, Spain	38.230	−6.030	8
R43	Umbria near Monchique, Portugal	37.335	−8.506	8

**TABLE 2:** Localities for the 32 study populations of marbled newts on the Lisbon peninsula, with sample sizes (*N*)

Population	Locality	Latitude	Longitude	*N*	Structure *Q*_m_ score
1	Salir de Matos*	39.427	−9.083	18	0.925
2	Fonte da Pena da Couvinha*	39.477	−9.019	2	0.936
3	Rio Maior*	39.340	−8.921	10	0
4	São Bartolomeu dos Galegos*	39.287	−9.280	6	0
5	Mosteiros de Alcanede*	39.426	−8.838	5	0
6	Casais Monizes*	39.449	−8.896	9	0
7	Santa Susana*	39.334	−9.002	2	0
8	Sintra 1	38.793	−9.417	2	0
9	Sintra 2	38.787	−9.388	2	0
10	Sintra Peninha	38.766	−9.460	30	0
11	Sintra 3	38.786	−9.428	12	0
12	Sintra 4	38.797	−9.390	1	0
13	Maceira	38.870	−9.309	15	0
14	Santo Estêvao das Galés	38.892	−9.247	6	0
15	Cabeço de Montachique	38.904	−9.187	13	0.002
16	Casas Novas	38.990	−9.266	14	0
17	Galegos	38.966	−9.211	13	0
18	Figueiredo	39.055	−9.234	15	0.001
19	Chanca	38.980	−9.300	15	0
20	Casais do Monte Bom	38.978	−9.373	5	0
21	Alvarinhos	38.891	−9.376	15	0
22	Casal da Serra	39.044	−9.371	14	0
23	Janas	38.827	−9.428	15	0
24	Portela	38.827	−9.263	15	0
25	Chãos	39.037	−9.316	15	0
26	Sobral de Monte Agraço	39.032	−9.138	14	0
27	Catém	39.080	−9.042	10	0
28	Cercal	39.225	−9.011	15	0
29	Valongo	39.171	−9.340	15	0
30	Vila Verde dos Francos	39.158	−9.137	5	0
31	Pinhôa	39.256	−9.238	15	0
32	Merceana	39.090	−9.120	11	0.001

Note

The right‐hand column presents the clustering assignment as obtained by Structure analysis under *K* = 2, with *Q*_m_ ranging from zero for *Triturus pygmaeus* to unity for *T. marmoratus*. Populations 1 and 2 are assigned as admixed and populations 3–32 are classified as *T. pygmaeus*. See also Figure 1. Localities with an asterisk have previously been studied by Espregueira Themudo and Arntzen (2007).

### 2.4 Population genetics

Hardy–Weinberg equilibrium and genotypic disequilibrium among pairs of nuclear loci were tested with the R package GENEPOP v1.0.5, under the Benjamini–Hochberg correction (Benjamini & Hochberg, 1995; Rousset, 2008). Gene flow between genetically distinct populations produces admixture linkage disequilibrium among those loci that have different allele frequencies in the founding populations (Pfaff et al., 2001). Admixture linkage disequilibrium was estimated following Barton and Gale (1993) and was based on 1,000 bootstrap replicates of the original dataset, using a published script (van Riemsdijk et al., 2019). We noted that the water bodies involved in significant instances of heterozygote deficit and admixture linkage disequilibrium had small dimensions (<2 m^3^) and presumably harbored small breeding populations, suggesting the possibility of sampling from within families. The STRING v.10.5 protein–protein interaction network database (Szklarczyk et al., 2015) was consulted to examine the functional linkage among the annotated nuclear markers, with reference to the *X. silurana* genome. Interactions were uncovered among nine marker pairs. However, these markers do not appear to be involved in significant deviations from Hardy–Weinberg equilibrium or in pairwise linkage disequilibrium. All markers were included in downstream analyses, as they appear to be physically and functionally unlinked in the populations investigated.

The SNP data were analyzed with Structure software under the “admixture model,” given that neighboring populations can interbreed (Falush et al., 2003; Pritchard et al., 2000). The program was run for 100,000 generations following 100,000 generations of burn‐in, with 10 replicates. The number of potentially differentiated gene pools was analyzed over the 1 < *K* < 10 range. The best *K* was selected using the “Evanno‐method” with StructureHarvester (Earl & vonHoldt, 2012). For results under *K* = 2, individuals were classified as pure *T. marmoratus* (*Q*_m_ > 0.95), pure *T. pygmaeus* (*Q*_m_ < 0.05) or genetically admixed (0.05 < *Q*_m_ < 0.95). Additionally, a principal component analysis (PCA) was performed on the SNPs dataset using Adegenet software (Jombart, 2008). Contour plots were made with MYSTAT (2007) software, with population sample size included as a weighing variable.

### 2.5 Environmental modeling

**FIGURE 3:**
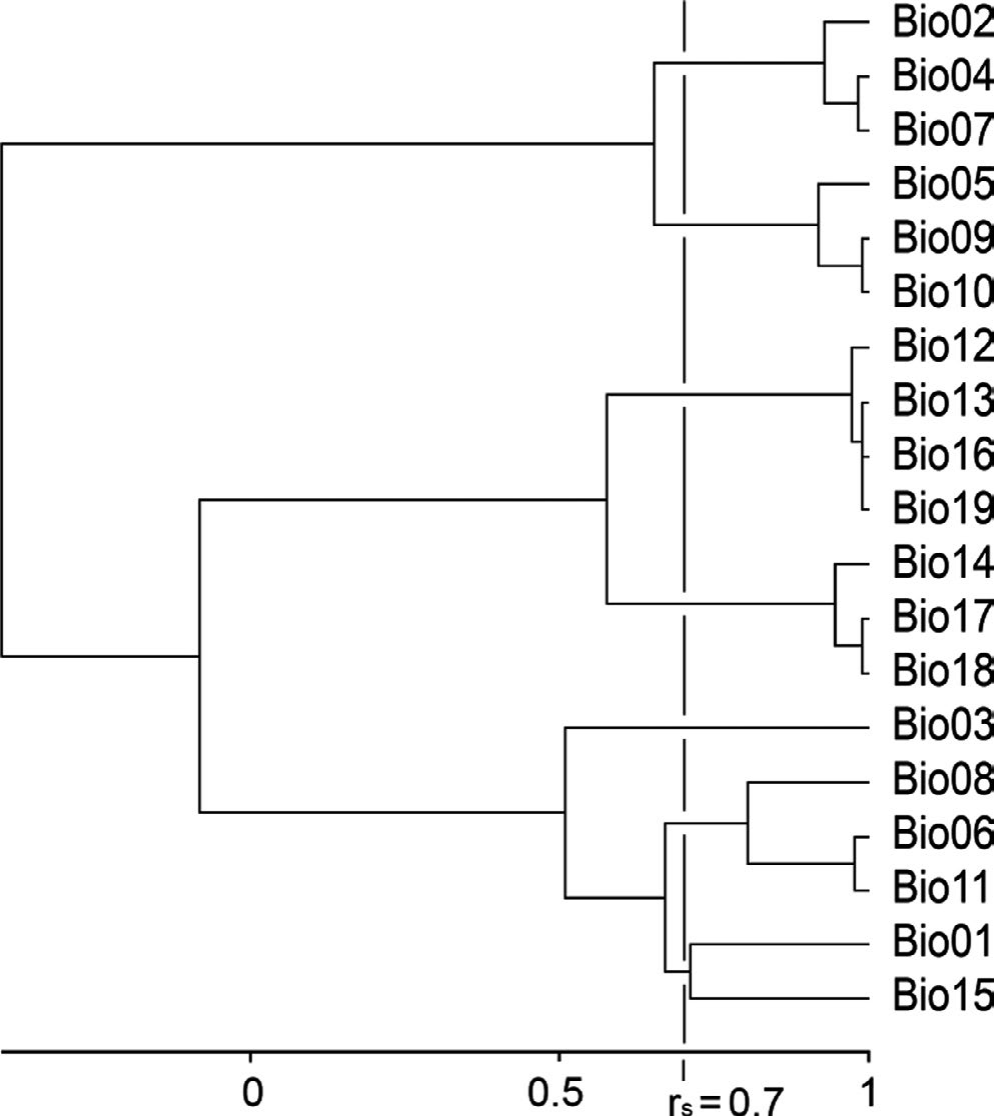
Analysis of collinearity for 19 climate variables from the BioClim 2.0 database extracted for 108 localities in the Iberian Peninsula with populations of *Triturus marmoratus* and *T. pygmaeus*. Variables selected for analysis using the criterion of partial independence at *r*_s_ < .7 were bio01, bio02, bio03, bio05, bio06, bio12, and bio17

A two‐species distribution model was constructed by the comparison of presence‐only data for both species under reference to contemporary climate conditions. The biological data employed were 108 *T. marmoratus* and *T. pygmaeus* records that were supported by molecular species identification (Arntzen, 2018; present paper). The records for three genetically admixed populations from Portugal and Spain and three *T. marmoratus* populations from France were excluded. Potential explanatory variables were the 19 climate parameters of WorldClim 2.0 (bio01‐bio19; see Fick & Hijmans, 2017). The pairwise Spearman's correlation coefficients (*r*_s_) were subjected to clustering with UPGMA in Primer‐e software (Clarke & Gorley, 2006). Variables were retained using the criterion of partial independence at *r*_s_ < .7 (Figure 3). Accordingly, variables selected for analysis were bio01, bio02, bio03, bio05, bio06, bio12, and bio17. Logistic regression analyses were performed with SPSS 20 (IBM SPSS, 2016) with “species” as the dependent variable. Parameter selection was in the forward stepwise mode under the criteria of *p*_in_ = 0.05 and *p*_out_ = 0.10 under the likelihood ratio criterion. Model fit was assessed by the area under the curve (AUC) statistic. The resulting distribution model was then applied to climate reconstructions for the Mid‐Holocene and the Last Glacial Maximum (WorldClim version 1.4; Hijmans et al., 2005). In the absence of firm guidance of which climate reconstruction would be most appropriate to apply (Guevara et al., 2019), distribution models were derived for all of them (i.e., nine models for the mid‐Holocene and three models for the Last Glacial Maximum). Distribution models were visualized with ILWIS (2009).

## 3 RESULTS

Single‐nucleotide polymorphisms were considered species diagnostic for 55 out of 64 nuclear and mitochondrial markers (Table S2). Significant deviations from Hardy–Weinberg equilibrium by heterozygote deficit were found four times in four populations, involving the loci BF, mrpl41, and sostdc1. Per‐population deviations were significant three times, in populations 10, 13, and 21. No significant pairwise linkage disequilibrium was detected per locus pair or population. Admixture linkage disequilibrium was significant in population 13. Protein functions were described for the 47 markers that could be annotated with the *X. tropicalis* genome (Table S3).

**FIGURE 4:**
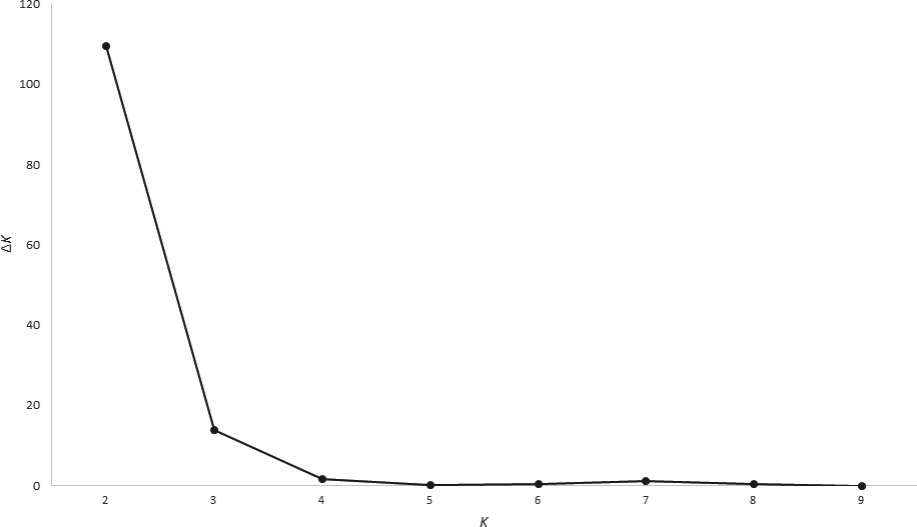
Estimation of the best number of *K* from an assumed range of 2–9 based on the Evanno method. ∆*K* was calculated as mean(|*L*″(*K*)|/sd(*L*(*K*))

In the Structure analysis, the number of genetically differentiated groups supported by the data was *K* = 2. Minor support was found for *K* = 3, and no support was obtained for Structure *K*‐values higher than that (Figure 4). Under *K* = 2, Structure classified 13 individuals from populations 1 and 2 (corresponding to the Caldas da Rainha enclave) as admixed (0.876 < *Q*_m_ < 0.949) and seven as *T. marmoratus* (0.954 < *Q*_m_ < 0.968). All other 334 individuals from populations 3–32 were classified as *T. pygmaeus* (*Q*_m_ < 0.019) (Figure 1c, Table 2). The SNP allele representing the *T. marmoratus* mtDNA haplotype was found in populations 1 and 2 and elsewhere only the *T. pygmaeus* haplotype was found. Allelic profiles consistent with genomic footprints were not observed. Accordingly, the genetic signature of *T. marmoratus* was restricted to the previously documented enclave where it displayed a low but notable level of introgression from *T. pygmaeus* into *T. marmoratus*.

**FIGURE 5:**
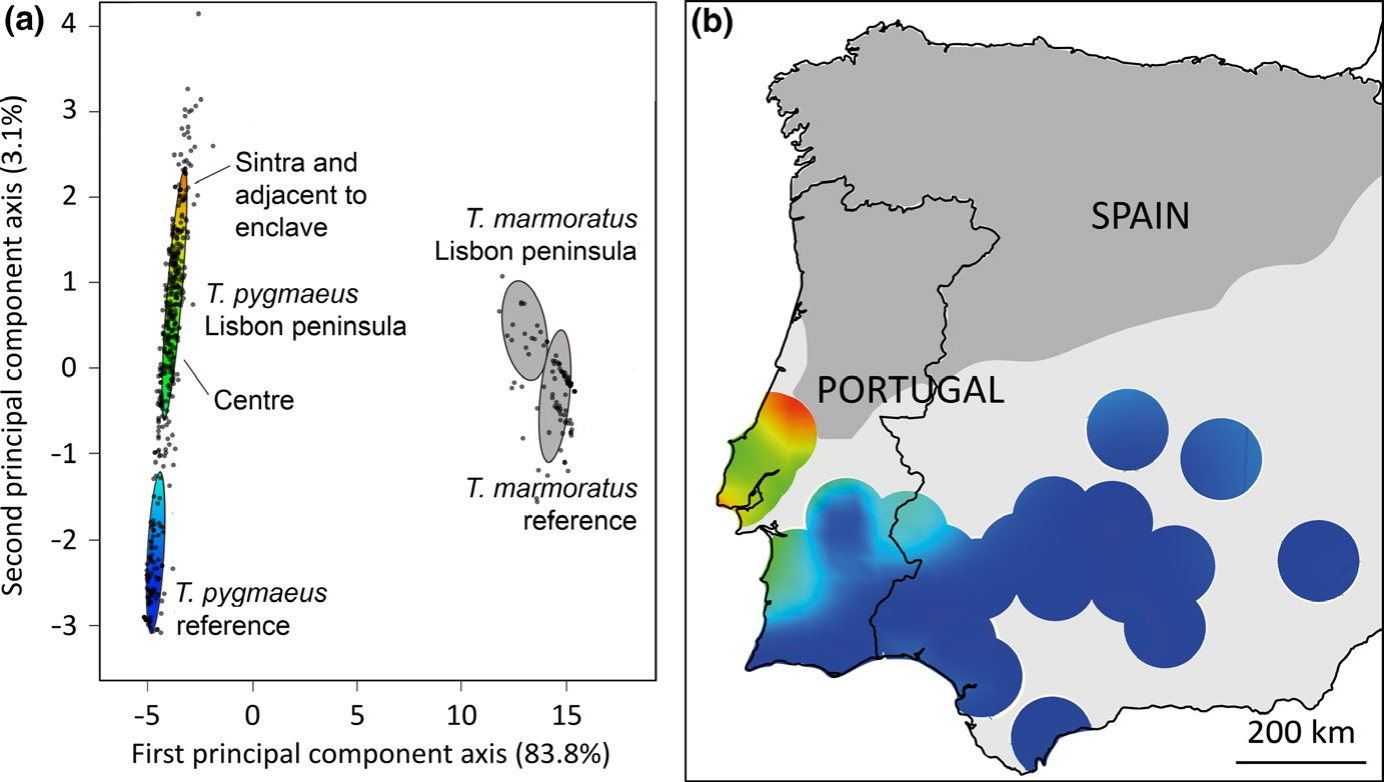
Principal component analysis of genetic variation at 54 biallelic nuclear loci in marbled newts from the Iberian Peninsula. (a) Results for *Triturus marmoratus* (gray shading) and *T. pygmaeus* (colors) for reference and Lisbon peninsula populations are summarized by ellipses that represent the mean ± one standard deviation. The amount of the total variation explained along the first and second PCA‐axis is 83.8% and 3.1%, respectively. (b) Contour plot of PCA2 scores for *T. pygmaeus* at the Lisbon peninsula. Results are shown for reference and Lisbon peninsula populations, with a per locality radius of ca. 100 km; see PCA2 color legend to the left. Note that Lisbon peninsula populations from the Serra de Sintra and adjacent to the *T. marmoratus* enclave have high scores (PCA2 > 1.5) and that centrally located populations have intermediate scores. Most reference populations have low scores (PCA2 < 1.5)

The first and second principal components of the PCA have eigenvalues that account for 83.8% and 3.1% of the total variance in the genetic data, respectively (Figure 5a). The third and higher axes contribute marginally to the explained variance and were not further considered. The first axis widely separates *T. marmoratus* and *T. pygmaeus*. Reference and Lisbon peninsula populations are also separated from one another, most strongly along the first axis in *T. marmoratus* and most strongly along the second axis in *T. pygmaeus*. The variation shown by *T. pygmaeus* is especially large, with uniformly negative PC2 axis values for reference samples from Spain and the south of Portugal and higher values for the Lisbon peninsula and for two populations that are geographically in between. A contour plot indicates that populations adjacent to the Caldas enclave and in the Serra de Sintra have higher scores than those at the center of the Lisbon peninsula (Figure 5b).

**FIGURE 6:**
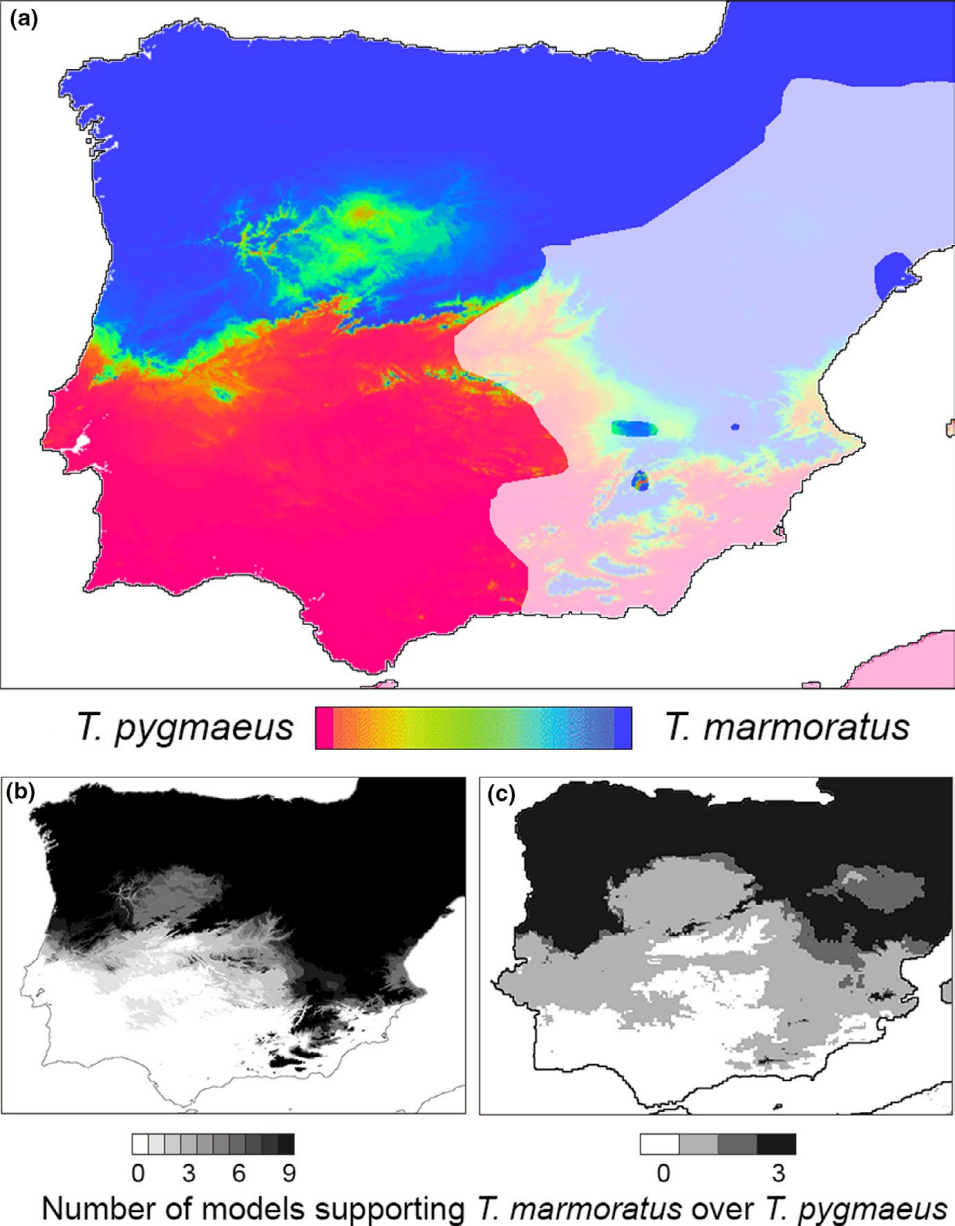
Two‐species distribution models for the newts *Triturus marmoratus* and *T. pygmaeus* over the Iberian Peninsula, derived from the climatic variable “precipitation of driest quarter.” Precipitation of the driest quarter is higher in the north and north‐western regions of the Iberian Peninsula. (a) Present day. The color legend shows the inferred probability for the presence of *T. marmoratus* *p*_m_ = 1 (blue) and *T. pygmaeus* (*p*_m_ = 0, red). Intermediate colors represent intermediate probabilities. The light shaded area falls outside the *Triturus* range (see Figure 1). (b) Distribution models over the western part of the Iberian Peninsula for the climate conditions of the Mid‐Holocene. Inferred species ranges are shown in gray (*p*_m_ > .5) and in white (*p*_m_ < .5). Model representation is binary and cumulative, so that the stepped gray scale represents the number of models supporting the presence of *T. marmoratus*, from zero to nine. (c) As in b, for three climate reconstructions at the Last Glacial Maximum. Note that most models support the contiguous species border to be more or less stable, whereas one model supports a more southern species border during the Last Glacial Maximum

The selected two‐species distribution model is represented by the logistic equation *p*_m_ = (1/(1 + exp(−0.156*bio17 + 7.767), in which *p*_m_ is the probability for the presence of *T. marmoratus* at the locality investigated, on a zero to unity scale and bio17 is “precipitation of driest quarter.” Model fit is AUC = 0.931 ± 0.025. The model describes more arid summer conditions for *T. pygmaeus* (mean precipitation over 60 localities is 36.4 ± 12.8 mm) than for *Triturus marmoratus* (mean precipitation over 48 localities is 67.4 ± 24.9 mm). The spatial interpretation of the model is shown in Figure 6a. Temporal extrapolations of the two‐species distribution models (or “hindcasts”) for the Mid‐Holocene are roughly similar to the one for the present (Figure 6a,b) whereas one out of three models for the Last Glacial Maximum support a more southerly located mutual species border (Figure 6a,c).

## 4 DISCUSSION

We employed a panel of presumably unlinked neutral markers to test for genetic traces of species replacement with hybridization in two species of marbled newts in Portugal. A northward hybrid zone movement has been proposed for this system, with a documented *T. marmoratus* enclave signaling the competitive advance of *T. pygmaeus*. We found no firm evidence for a genetic footprint of *T. marmoratus* in *T. pygmaeus* in the Lisbon peninsula. Analysis with Structure showed the unequivocal signal for interspecific gene flow (Figure 1c), but this was limited to the *T. marmoratus* enclave, in line with earlier observations (Espregueira Themudo & Arntzen, 2007). This result was not impacted by the significant instances of heterozygote deficit and admixture linkage disequilibrium, which were likely due to the relatedness among the sampled larvae in small ponds (cf. Goldberg & Waits, 2010). We found no evidence for any additional enclave or species admixture. This result contrasts that of a pilot study where we observed one individual with admixed genetic characteristics in the Serra de Sintra (Arntzen et al., in press). The two individuals from this population included in the present study classify as *T. pygmaeus*, which could stem from the more restrictive nature of the present data than the pilot study's Ion Torrent sequencing data. Therefore, we cannot conclude on the genetic characteristics of the Serra de Sintra population.

The PCA analysis positioned *T. marmoratus* from the Lisbon peninsula enclave away from reference *T. marmoratus* and in the direction of the local *T. pygmaeus* populations (Figure 5a). We interpret the spatial context of introgressed genes as an indication of the start of genetic erosion of the *T. marmoratus* enclave. We expect the enclave to eventually disappear under *T. pygmaeus*' competitive advance, leading to the loss of *T. marmoratus* gene variants over time. It is tempting to attribute the unexpected genetic differentiation within *T. pygmaeus* to gene flow from *T. marmoratus*. The *T. pygmaeus* populations in Serra de Sintra and “adjacent to the enclave” are those where the differentiation is most pronounced, and are positioned exactly where we would expect introgression to have taken place. This interpretation is, however, not supported by the PCA analysis in which the second axis appears to differentiate intraspecific variation (i.e., within *T. pygmaeus*), independent from the first axis that appears to differentiate interspecific differentiation (i.e., vs. *T. marmoratus*). An alternative explanation is the presence of geographical variation, in which *T. pygmaeus* populations from Spain and the south of Portugal are different from those more to the northwest. It must be noted that our pilot study also showed substantial variation within *T. pygmaeus* (Arntzen et al., in press).

We identified the parameter “amount of precipitation in the driest quarter of the year” (bio17) as most closely and significantly associated with the two‐species distributions (Figure 6). Newts of *T. pygmaeus* thrive in wide, shallow and ephemeral water bodies, whereas *T. marmoratus* fares better in smaller, deeper and more permanent breeding sites and under moister terrestrial conditions (Espregueira Themudo & Arntzen, 2007). The driver of the inferred species turnover may have been dwindling levels of precipitation, giving *T. pygmaeus* a competitive advantage over its sister species, especially along the coast line. Given that *Triturus* species may disperse at 1 km a year (Kupfer, 1998; Kupfer & Kneitz, 2000; Trochet et al., 2014), the inferred 200 km range extension could be achieved in a couple of centuries, even when competition with a related species slows down the advance (Arntzen & Wallis, 1991; Wielstra et al., 2017). The reconstructed paleo‐climatic data yield little support for a wider, more southerly distribution of *T. marmoratus* in the Mid‐Holocene (Figure 6b) and for the Last Glacial Maximum the support is limited to one climate reconstruction out of the three that are available (Figure 6c). The advancement of *T. pygmaeus* over *T. marmoratus* may thus have been more recent than the climate data we analyzed. Whether or not the hybrid zone movement is still going on may be a compelling reason to continue monitoring.

The absence of a genomic footprint on the Lisbon peninsula in the current study suggests that the *T. marmoratus* pocket may have originated through natural or anthropogenic dispersal. However, these are unlikely explanations, as the gap separating the enclave from the main range is beyond the direct dispersal capacity of *Triturus* newts, and there is no tradition of newt husbandry with possible translocations in Portugal. Therefore, two main biogeographic scenarios of enclave formation arise in the absence of a clear genomic footprint in the Lisbon peninsula: (a) The species could have undergone negligible introgression or (b) displacement could have occurred with introgressive hybridization, but with the signal subsequently lost.

Firstly, species replacement may have taken place on the Lisbon Peninsula without (detectable) hybridization. Prezygotic barriers, such as mating preference or genetic incompatibility, as well as postzygotic effects, including Dobzhansky–Muller incompatibilities, are known to occur in *Triturus* newts, and could be limiting hybridization among the species (Arntzen et al., 2009; Coughlan & Matute, 2020; Zuiderwijk, 1990). Hybridization between *T. marmoratus* and *T. pygmaeus* is not frequent, as is illustrated by the narrow characteristics of the hybrid zone for many characters (Arntzen, 2018), as well as by the local population Juncal (Figure 1), where species composition is bimodal (two hybrids were found along with twelve *T. marmoratus* and two *T. pygmaeus*; Espregueira Themudo & Arntzen, 2007). Variants of this scenario are the retraction of *T. marmoratus* followed by expansion of parapatric *T. pygmaeus* into abandoned ponds, or that hybridization and introgression are too infrequent to be detected with the current biological and genetic sampling schemes. Transcriptomic‐derived markers may not be selectively neutral and possibly fail to detect asymmetric introgression; therefore, we performed tests to identify any markers under selection and concluded our markers displayed neutral behavior. An argument against this explanation is that *T. marmoratus* and *T. pygmaeus* do, at the present day, hybridize along the species contact zone (Arntzen, 2018; Arntzen et al., in press).

Secondly, species replacement may have occurred with hybridization. The genetic differentiation of *T. pygmaeus* in the Lisbon peninsula could be attributed to hybridization and purifying selection, with introgressed alleles removed at some loci and not others. Strong selection against hybrids (purifying selection), as suggested by the low numbers of admixed individuals reported so far, could have eroded the footprint. In support of this scenario is that simulation models suggest that narrow (bimodal) hybrid zones—as observed for *T. marmoratus* and *T. pygmaeus*—are consistent with strong selection against hybrids (Currat et al., 2008; Singhal & Moritz, 2011). If the force of selection is sufficient, only sporadical take‐up of advantageous alleles by the invading species may take place, as has been previously shown for toads (*Bufo*) in the UK (Arntzen, 2019). Such a scenario would be comparable to the phenomenon of “leaky replacement” of Neanderthals by other hominins (Hedrick, 2013; Pardo‐Diaz et al., 2012; Racimo et al., 2015).

Both biogeographic scenarios of species replacement are subject to the unknown past position of the, presumably parapatric, *T. marmoratus—T. pygmaeus* species boundary. Environmental modeling suggests *T. marmoratus* might not have inhabited the Lisbon Peninsula in the past (Figure 6b,c), so species replacement could have been limited to the current, circa 5 km wide distribution gap. A detailed search for genetic footprints in the area surrounding the enclave may help to differentiate the most likely among the proposed scenarios. The replacement of *T. marmoratus* by *T. pygmaeus* is possibly adversely influenced by the loss of breeding sites in southern Spain and Portugal, where temporary ponds are in decline because of agricultural intensification, desertification, and climate warming (Thomas et al., 2006; van de Vliet et al., 2014). This would parallel observations on another *Triturus* moving hybrid zone that was shown to have halted due to the loss of breeding sites (Visser et al., 2017). Understanding shifts in species distributions, particularly when driven by climate change and anthropogenic activities, are especially relevant when deciphering the dynamics of species replacement (Taylor et al., 2015).

## CONFLICT OF INTEREST

The authors declare no competing interests.

## AUTHOR CONTRIBUTIONS

**Julia López‐Delgado:** Conceptualization (supporting); Data curation (lead); Formal analysis (lead); Funding acquisition (supporting); Investigation (lead); Methodology (equal); Writing‐original draft (lead); Writing‐review & editing (equal). **Isolde van Riemsdijk:** Formal analysis (supporting); Investigation (supporting); Methodology (equal); Supervision (equal); Writing‐review & editing (equal). **Jan W. Arntzen:** Conceptualization (lead); Formal analysis (supporting); Funding acquisition (lead); Investigation (supporting); Methodology (equal); Supervision (equal); Writing‐review & editing (equal).

## Supporting information

Table S1‐S3

## Data Availability

Supplemental tables are available in: EcolEvol_Supplementary_Tables_López‐Delgado_2020.xlsx (Supporting Information).
